# Internet-Based Abnormal Chromosomal Diagnosis During Pregnancy Using a Noninvasive Innovative Approach to Detecting Chromosomal Abnormalities in the Fetus: Scoping Review

**DOI:** 10.2196/58439

**Published:** 2024-10-16

**Authors:** Mega Obukohwo Sr Oyovwi, Ejiro Peggy Ohwin, Rume Arientare Rotu, Temitope Gideon Olowe

**Affiliations:** 1 Department of Physiology Adeleke University Ede Nigeria; 2 Department of Human Physiology Faculty of Basic Medical Science Delta State University Abraka Nigeria; 3 Department of Physiology, University of Ibadan Ibadan Nigeria; 4 Department of Obstetrics & Gynaecology University of Medical Sciences Ondo Nigeria

**Keywords:** internet-based, abnormal chromosomal diagnosis, pregnancy, noninvasive, innovative approach, detecting, preventing, chromosomal abnormalities, fetus

## Abstract

**Background:**

Chromosomal abnormalities are genetic disorders caused by chromosome errors, leading to developmental delays, birth defects, and miscarriages. Currently, invasive procedures such as amniocentesis or chorionic villus sampling are mostly used, which carry a risk of miscarriage. This has led to the need for a noninvasive and innovative approach to detect and prevent chromosomal abnormalities during pregnancy.

**Objective:**

This review aims to describe and appraise the potential of internet-based abnormal chromosomal preventive measures as a noninvasive approach to detecting and preventing chromosomal abnormalities during pregnancy.

**Methods:**

A thorough review of existing literature and research on chromosomal abnormalities and noninvasive approaches to prenatal diagnosis and therapy was conducted. Electronic databases such as PubMed, Google Scholar, ScienceDirect, CENTRAL, CINAHL, Embase, OVID MEDLINE, OVID PsycINFO, Scopus, ACM, and IEEE Xplore were searched for relevant studies and articles published in the last 5 years. The keywords used included *chromosomal abnormalities*, *prenatal diagnosis*, *noninvasive*, and *internet-based*, and *diagnosis*.

**Results:**

The review of literature revealed that internet-based abnormal chromosomal diagnosis is a potential noninvasive approach to detecting and preventing chromosomal abnormalities during pregnancy. This innovative approach involves the use of advanced technology, including high-resolution ultrasound, cell-free DNA testing, and bioinformatics, to analyze fetal DNA from maternal blood samples. It allows early detection of chromosomal abnormalities, enabling timely interventions and treatment to prevent adverse outcomes. Furthermore, with the advancement of technology, internet-based abnormal chromosomal diagnosis has emerged as a safe alternative with benefits including its cost-effectiveness, increased accessibility and convenience, potential for earlier detection and intervention, and ethical considerations.

**Conclusions:**

Internet-based abnormal chromosomal diagnosis has the potential to revolutionize prenatal care by offering a safe and noninvasive alternative to invasive procedures. It has the potential to improve the detection of chromosomal abnormalities, leading to better pregnancy outcomes and reduced risk of miscarriage. Further research and development in this field is needed to make this approach more accessible and affordable for pregnant women.

## Introduction

### Background

Prenatal diagnosis of chromosomal abnormalities is an important part of prenatal care. Chromosomal abnormalities are the major cause of pregnancy complications, including miscarriage, stillbirth, and birth defects [1]. Understanding the prevalence and impact of commonly diagnosed chromosomal abnormalities in pregnancy is essential for providing accurate genetic counseling and appropriate prenatal care. Traditionally, prenatal diagnosis has been performed using invasive methods such as amniocentesis and chorionic villus sampling. However, these methods are associated with a small risk of miscarriage [2,3]. In recent years, noninvasive prenatal testing (NIPT) has emerged as a safe and effective alternative to invasive methods. NIPT is based on the analysis of cell-free DNA (cfDNA) in the maternal blood [3]. cfDNA is released into the maternal blood by the placenta and contains genetic material from both the mother and the fetus. This advent of NIPT has revolutionized prenatal diagnosis [3]. While NIPT has emerged as a powerful tool for detecting common chromosomal abnormalities such as Down syndrome, its accessibility and potential for broader application through internet-based platforms remain relatively unexplored. This review focuses on understanding the feasibility, benefits, and challenges of using internet-based technologies to deliver NIPT services effectively. Internet-based NIPT presents a compelling opportunity to overcome barriers associated with traditional prenatal diagnostics [4]. Web-based platforms can extend NIPT services to geographically remote areas and underserved populations, bridging health care disparities. Web-based platforms offer flexible scheduling and internet consultations, reducing the need for multiple clinic visits, especially beneficial for working mothers [5]. Internet-based platforms can potentially streamline administrative processes and reduce operational costs, making NIPT more affordable for a wider population [5]. This review aims to provide a comprehensive overview of the current state of internet-based NIPT, discussing its technical feasibility, ethical considerations, and potential impact on prenatal care. Notwithstanding, current prenatal chromosomal diagnosis methods have several limitations. They are invasive, expensive, and can cause anxiety in pregnant women. Therefore, there is a need for internet-based abnormal chromosomal diagnosis, a noninvasive, cost-effective, and anxiety-reducing method for chromosomal abnormality detection.

Internet-based methods for prenatal diagnosis of chromosomal abnormalities are becoming increasingly popular. These methods allow pregnant women to access information and support from health care professionals and other parents who have experienced similar challenges [6]. There are a number of different internet-based methods for prenatal diagnosis, including web-based genetic counseling, online support groups, and web-based prenatal testing [7-9]. Internet-based abnormal chromosomal diagnosis during pregnancy is a noninvasive and innovative approach to detecting chromosomal abnormalities in fetuses, offering several advantages over traditional invasive procedures [5]. This review aims to provide a comprehensive overview of this emerging technique, highlighting its benefits, limitations, and implications for prenatal care. Moreover, internet-based abnormal chromosomal diagnosis during pregnancy aims to address these limitations by using advanced computational techniques to analyze fetal genetic material obtained through noninvasive methods, such as maternal blood samples. This approach offers a safe and convenient alternative to traditional invasive procedures. This review aims to provide a comprehensive understanding of internet-based abnormal chromosomal diagnosis during pregnancy. By exploring this emerging technology, we can contribute to improving the safety, accessibility, and effectiveness of prenatal chromosomal abnormality detection.

### Basics of Chromosomal Abnormalities

**Figure 1:**
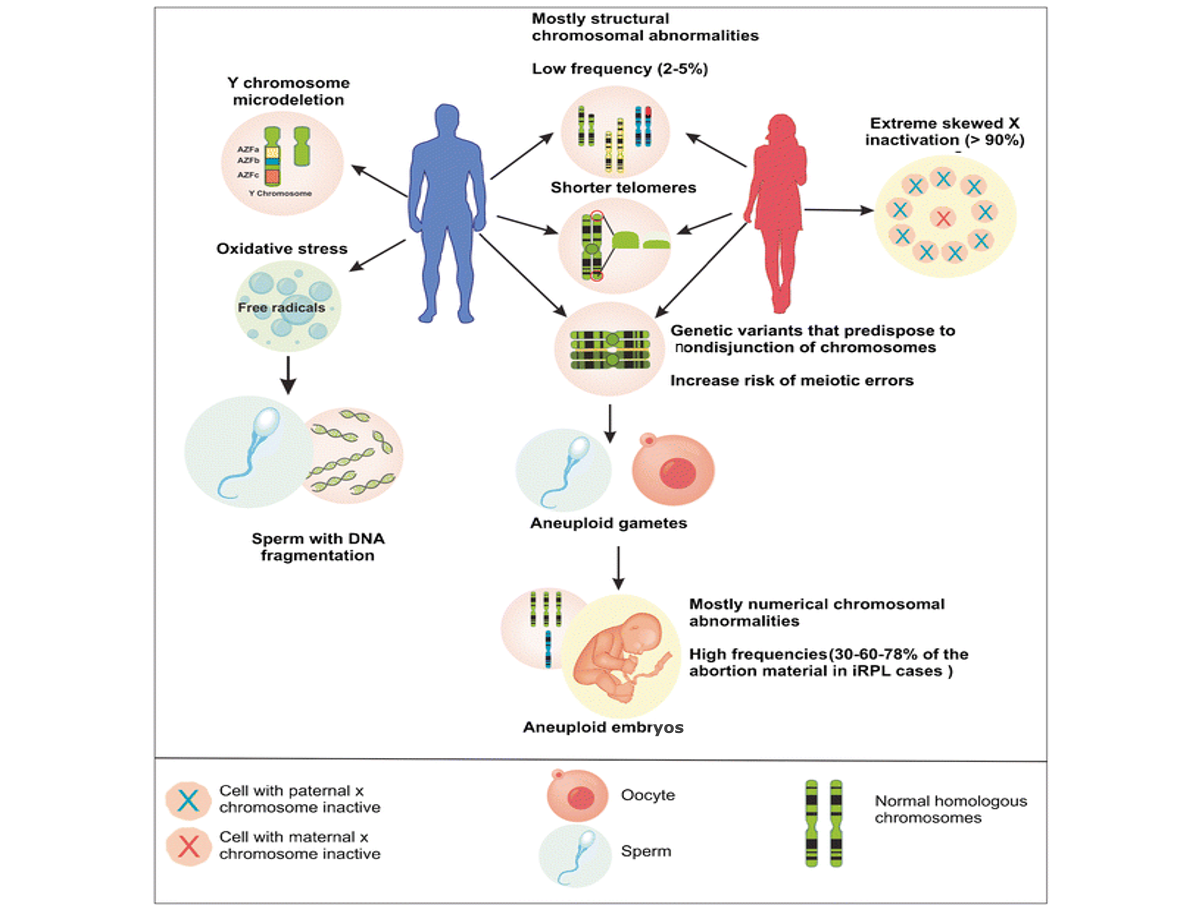
Chromosomal abnormalities linked to repeated miscarriages. There is evidence linking chromosomal anomalies to repeated miscarriages at the parent, gamete, and fetal levels. Abnormalities in numbers and structure provide the most compelling evidence of a connection to the illness. iRPL: idiopathic recurrent pregnancy loss.

Chromosomal abnormalities involve changes in the number or structure of chromosomes, which contain genetic information determining physical traits [10]. These can lead to health issues such as developmental delays, birth defects, and genetic disorders (Figure 1 [10]). There are 2 main types of abnormalities: numerical and structural [11]. Numerical abnormalities involve whole chromosome loss, while structural abnormalities involve chromosome structure changes [12] (Textbox 1).

**Numerical and structural abnormalities.**

**Numerical abnormalities**

- Aneuploidy: aneuploidy is a condition where there is an abnormal number of chromosomes in the cells. The most common examples of aneuploidy include trisomy (an extra copy) and monosomy (a missing copy) of a chromosome. The most well-known example of aneuploidy is Down syndrome, which is caused by an extra copy of chromosome 21 [13].
- Polyploidy: polyploidy refers to the presence of >2 sets of chromosomes in a cell. It is relatively rare in humans, but it can lead to severe birth defects and developmental delays [14].

**Structural abnormalities**

- Deletion: a deletion occurs when a part of a chromosome is missing or deleted. This can result in the loss of essential genetic information and can lead to various health issues, including physical and cognitive disabilities [15].
- Duplication: duplication is when a section of a chromosome is duplicated, resulting in an extra copy of genetic material [16]. Duplication can lead to developmental delays, intellectual disabilities, and other health problems.
- Translocation: translocation occurs when a part of one chromosome breaks off and attaches to another chromosome [17]. This can result in a rearrangement of genetic material and can cause various health issues depending on the genes involved.

### Causes and Risk Factors

Chromosomal abnormalities can occur due to various causes [18], including (1) genetic inheritance: some chromosomal abnormalities can be inherited from one or both parents, such as Down syndrome, which is caused by an extra copy of chromosome 21 inherited from the mother or father; (2) errors in cell division: chromosomal abnormalities can also occur during the process of cell division, for example, an error in the division of sex cells (eggs and sperm) can result in an embryo with an abnormal number of chromosomes; and (3) exposure to environmental factors: exposure to certain environmental factors, such as radiation, chemicals, and toxins, can increase the risk of chromosomal abnormalities in pregnancy.

## Methods

### Search Strategy

To comprehensively explore the landscape of chromosomal abnormalities and noninvasive prenatal diagnosis and therapy, a thorough literature review was undertaken. This review encompassed a wide range of electronic databases including PubMed, Google Scholar, ScienceDirect, CENTRAL, CINAHL, Embase, OVID MEDLINE, OVID PsycINFO, Scopus, ACM, and IEEE Xplore (Multimedia Appendix 1). The search focused on studies and articles published within the last 5 years, using keywords such as *chromosomal abnormalities*, *prenatal diagnosis*, *noninvasive*, and *internet-based approach*. This multifaceted search strategy aimed to capture the most relevant and current research on this topic. The search was further refined by applying filters for language (English), publication type (journal articles, systematic reviews, and meta-analyses), and time frame (from database inception to the present). In addition, reference lists of retrieved articles and relevant textbooks were manually inspected for additional pertinent studies. This comprehensive search strategy ensured the identification of a wide range of literature exploring the internet-based abnormal chromosomal diagnosis during pregnancy: a noninvasive innovative approach to detecting chromosomal abnormalities in the fetus, thus providing a robust foundation for this review.

### Inclusion and Exclusion Criteria

The inclusion criteria for this review were studies that focused on chromosomal abnormalities and internet-based diagnosis. Studies that used an internet-based approach to detect and quantify chromosomal abnormalities in the fetus were also included. The exclusion criteria were studies that did not focus on chromosomal abnormalities or did not have a specific focus on internet-based approaches. Studies that were not published in the English language or were published before 2000 were also excluded.

### Ethics Approval

This review was conducted in accordance with the guidelines and approval of the Research, Ethics, and Grants Committee of the Faculty of Basic Medical Sciences, Adeleke University, Ede, Nigeria.

## Results and Discussion

### Internet-Based Abnormal Chromosomal Diagnosis

#### Overview

**Figure 2:**
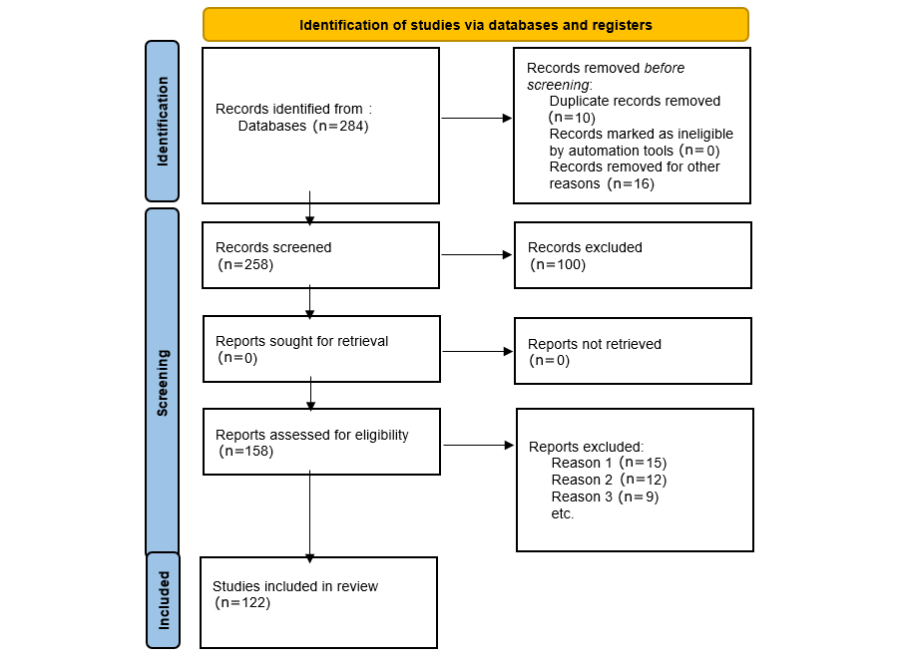
PRISMA (Preferred Reporting Items for Systematic Reviews and Meta-Analyses) flowchart. Reason 1 (n=15): studies not providing data specifically related to noninvasive methods for detecting chromosomal abnormalities in the fetus. Reason 2 (n=12): reports that were not focused on internet-based or telehealth approaches in delivering prenatal diagnosis or genetic counseling. Reason 3 (n=9): articles lacking peer-reviewed status, including nonscientific articles, opinion pieces, or conference abstracts that did not meet rigorous research standards.

Figure 2 shows an overview of the included studies. The rapid advancements in technology have transformed the field of medicine, including the way we diagnose and treat diseases. One such groundbreaking approach is internet-based abnormal chromosomal diagnosis. This approach uses the internet to provide genetic counseling and testing for individuals with abnormal chromosomal conditions. Here, we discuss the definition and explanation of this approach as well as how it works through genetic counseling and testing via web-based platforms and kits.

#### Definition and Explanation of the Approach

Internet-based abnormal chromosomal diagnosis is a novel approach in which individuals with abnormal chromosomal conditions can receive genetic counseling and testing through web-based platforms [19]. This approach uses the internet to provide individuals with access to genetic counseling and testing services without the need to physically visit a health care facility [19]. Genetic counselors, which are health care professionals trained in genetics, use web-based platforms to communicate with patients and provide them with information about their condition, potential risks, and available treatment options. This approach also offers genetic testing kits that can be used at home to collect samples, which are then sent to a laboratory for analysis.

#### Decoding DNA: A Guide to Web-Based Genetic Testing and Counseling

##### Genetic Counseling Through Web-Based Platforms

The advent of internet-based technologies has revolutionized the delivery of health care services, including genetic counseling. Scientific research has explored the effectiveness and benefits of genetic counseling through web-based platforms, offering valuable insights into the transformative potential of this approach [20]. Web-based genetic counseling involves using virtual communication tools, such as videoconferencing and secure messaging, to provide genetic risk assessment, education, and support to individuals and families seeking genetic information [21].

Studies have demonstrated the efficacy of web-based genetic counseling in providing accurate and comprehensive genetic information [22]. Through secure and Health Insurance Portability and Accountability Act–compliant platforms, genetic counselors effectively collect family and medical histories, interpret genetic test results, and discuss inheritance patterns and risk implications [23-25]. Research has also shown that web-based genetic counseling is noninferior to in-person counseling in terms of patient satisfaction, knowledge acquisition, and decision-making [26]. Moreover, web-based platforms can overcome geographical barriers, allowing individuals in remote or underserved areas to access specialized genetic counseling services.

The convenience and flexibility of web-based genetic counseling have gained traction among patients. Studies indicate that individuals appreciate the ability to schedule appointments at their convenience, eliminate travel time and expenses, and access genetic counseling from the comfort of their own homes [27]. Web-based platforms also offer greater accessibility for individuals with mobility issues, chronic health conditions, or busy schedules.

Research has further highlighted the cost-effectiveness of web-based genetic counseling. By reducing the need for in-person visits and travel expenses, web-based platforms can make genetic counseling more accessible and affordable for patients [28]. This can be particularly impactful for individuals facing financial constraints or those living in areas with limited access to genetic services.

##### Genetic Testing Through Web-Based Kits

Genetic testing through web-based kits has gained significant popularity in recent years, offering individuals the opportunity to learn about their genetic makeup and potential health risks. However, the accuracy and reliability of these tests have been subject to scientific scrutiny. Several studies have evaluated the performance of web-based genetic testing kits and have reported mixed results. In a study, web-based genetic testing kits have been proven to provide individuals with a convenient and accessible way to collect and submit their DNA samples for analysis [29]. Some studies have found that these kits can provide accurate and reliable information about certain genetic markers, while others have raised concerns about their limitations [30]. For example, a study found that a web-based genetic testing kit was able to accurately identify the presence of the *Breast cancer gene 1* (*BRCA*) mutation, which increases the risk of breast and ovarian cancer, with high sensitivity and specificity [31]. However, another study reported that several web-based genetic testing kits produced inaccurate results for certain genetic variants, particularly those associated with rare diseases [32]. These findings suggest that the accuracy and reliability of web-based genetic testing kits can vary depending on the specific genetic markers being tested and the quality of the laboratory performing the analysis.

#### Internet-Based Models of Chromosomal Abnormality Diagnosis and Performance Metrics

##### Overview

Internet-based models of chromosomal abnormality diagnosis have become increasingly common in recent years. These models use advanced technologies and algorithms to analyze genetic data and identify potential chromosomal abnormalities in patients [7-9]. These models use advanced algorithms to analyze genetic data and identify potential abnormalities, which can then be further analyzed by medical professionals. This allows faster diagnosis and treatment, which can be critical for patients with serious genetic conditions. In terms of performance metrics, internet-based models are typically evaluated based on their accuracy, speed, and cost-effectiveness. Accuracy is a critical metric because it directly impacts patient outcomes. Studies have shown that internet-based models are highly accurate in detecting chromosomal abnormalities, with some models reporting 99% accuracy rates [33]. Speed is also an important performance metric, as a faster diagnosis can lead to earlier treatment and better outcomes for patients. Internet-based models are able to analyze large amounts of data in a fraction of the time it would take for traditional methods, allowing for faster diagnosis and treatment [34,35]. Cost-effectiveness is another key metric for evaluating internet-based models. These models are typically more affordable than traditional methods, making them accessible to a wider range of patients. In addition, the use of internet-based models can reduce the need for expensive and invasive diagnostic procedures, further reducing costs [36].

##### Virtual Karyotyping

Virtual karyotyping is an internet-based model for chromosomal abnormality diagnosis that uses high-resolution imaging and computer algorithms to generate a digital representation of an individual’s chromosomes [37]. This method allows the detection of chromosomal abnormalities, such as deletions, duplications, and translocations, without the need for traditional chromosome analysis techniques. This method processes digital images of chromosomes obtained through various techniques such as fluorescence in situ hybridization or spectral karyotyping to generate a virtual representation of the karyotype. The performance metrics for virtual karyotyping include sensitivity and specificity, which measure the accuracy of the test in detecting true positive and true negative results, respectively. The review of numerous studies reveals that virtual karyotyping significantly enhances the speed, accuracy, and efficiency of chromosomal analysis. It allows automated chromosome identification and banding pattern analysis, eliminating subjective interpretation and reducing human error [38]. This automation also facilitates the analysis of large datasets, which is particularly crucial for population-based studies and screening programs. Furthermore, virtual karyotyping offers advantages in terms of cost-effectiveness and flexibility. The elimination of physical chromosome preparation and analysis reduces the overall cost and time associated with traditional karyotyping. Moreover, the digital nature of virtual karyotyping allows easy data storage, sharing, and analysis, making it readily accessible for research and clinical applications. Notably, virtual karyotyping has proven its value in identifying chromosomal abnormalities associated with genetic disorders, including aneuploidy, translocations, and deletions. Its ability to detect subtle chromosomal alterations that might be missed in conventional karyotyping further enhances its diagnostic power.

#### Next-Generation Sequencing

Next-generation sequencing (NGS) has revolutionized biological research, enabling the rapid and cost-effective sequencing of entire genomes, exomes, and transcriptomes [39]. This technology has spurred a surge in scientific studies across various fields, ranging from human disease research to evolutionary biology and environmental science. NGS platforms, such as Illumina, Ion Torrent, and PacBio, offer distinct advantages, including high throughput, increased sensitivity, and the ability to identify rare variants [40]. Illumina (Illumina Inc) is a top NGS platform with high throughput and accuracy, offering software tools like BaseSpace Sequence Hub, DRAGEN Bio-IT Platform, Real-Time Analysis, and Illumina Connected Analytics for data storage, analysis, and population-wide studies. Ion Torrent (Thermo Fisher Scientific), a semiconductor-based sequencing technology, offers software tools like Ion Suite, Ion Reporter Software, and Torrent Suite Software for data analysis, variant interpretation, and workflow management. PacBio’s (Pacific Biosciences of California Inc) SMRT Analysis Software offers tools for analyzing long-read data, genome assembly, and error correction, while its Bioinformatics Software offers genome assembly and error correction applications. Circular Consensus Sequencing enhances accuracy by generating consensus sequences. Bioinformatics tools like CLC Genomics Workbench, Partek Genomics Suite, and GensearchNGS are compatible with multiple NGS platforms, enhancing their versatility and integrating microarray data with NGS applications. Hence, Studies using NGS have led to significant advancements in our understanding of genetic diseases, cancer biology, and microbial diversity [41]. For instance, whole-genome sequencing has facilitated the identification of disease-causing mutations, while RNA sequencing has shed light on gene expression patterns and regulatory mechanisms. Furthermore, NGS has facilitated the development of personalized medicine approaches tailored to individual genetic profiles. However, NGS data analysis presents significant challenges, requiring specialized bioinformatics expertise and powerful computational resources. Performance metrics for NGS include sensitivity, specificity, and positive predictive value, which measures the proportion of positive results that are true positives.

#### Microarray Analysis

Microarray analysis is an internet-based model for chromosomal abnormality diagnosis that uses DNA microarrays to detect copy number variations (CNVs) and other chromosomal abnormalities [42]. This method is particularly useful for detecting small deletions and duplications that may not be visible using traditional chromosome analysis techniques. Array-based technologies have revolutionized our ability to study the human genome. These technologies allow for high-throughput analysis of genetic variation and have been instrumental in identifying genetic markers associated with disease susceptibility [42]. Studies using microarray analysis have yielded significant insights into diverse fields, including disease mechanisms, drug discovery, and personalized medicine [43]. The process typically involves extracting RNA from samples, converting it to complementary DNA, and hybridizing the complementary DNA to a microarray chip containing thousands of probes corresponding to specific genes. By measuring the intensity of the fluorescent signal emitted from each probe, researchers can quantify the relative expression levels of genes in different experimental conditions. This high-throughput approach has enabled the identification of gene signatures associated with various diseases, such as cancer and neurodegenerative disorders, providing valuable information for diagnosis, prognosis, and treatment development.

One type of variation that has been of particular interest is CNV, which refers to the presence of an abnormal number of copies of a specific DNA segment in the genome. CNVs can range in size from a few hundred base pairs to several megabases and have been shown to play a significant role in human diseases, including cancer, neurological disorders, and developmental disorders. Several array-based technologies have been developed for CNV detection, including comparative genomic hybridization arrays, single-nucleotide polymorphism (SNP) arrays, and oligonucleotide arrays [42]. SNP arrays, in particular, have become a popular tool for CNV detection due to their ability to simultaneously genotype and detect CNVs [42]. One such SNP array technology is the BeadArray platform, which is developed by Illumina Inc. This technology uses bead-based arrays to interrogate >1 million SNPs across the human genome [42]. While SNP arrays have been successful in detecting CNVs, there is still a need for improved computational tools for accurate and high-resolution CNV detection. In recent years, there has been a growing interest in developing objective Bayesian methods for CNV detection, as these methods allow for more robust and accurate statistical inference. In this paper, we discuss the development and validation of a novel computational framework, QuantiSNP, for CNV detection using BeadArray SNP genotyping data.

QuantiSNP is a novel computational framework for high-resolution CNV detection from BeadArray SNP genotyping data. It uses an objective Bayes hidden-Markov model and incorporates objective Bayesian measures and maximum marginal likelihood to set model parameters. The algorithm has been experimentally validated and shown to significantly improve the accuracy of aneuploidy identification and mapping compared to existing analytical tools [42]. It is a versatile tool that can be adapted to other platforms and has widespread applicability in genomic research, particularly in the fields of clinical genetics, cancer, and disease association studies. With the increasing use of array-based technologies in genetic research, QuantiSNP has the potential to make a significant impact in understanding the role of CNVs in human diseases. The performance metrics for microarray analysis include sensitivity, specificity, and positive predictive value.

#### Bioinformatics Tools

Bioinformatics tools are internet-based models for chromosomal abnormality diagnosis that use complex algorithms to analyze genetic data and identify potential chromosomal abnormalities [43]. These tools can be used in conjunction with other diagnostic methods, such as karyotyping or NGS, to improve the accuracy and efficiency of chromosomal abnormality diagnosis. The performance metrics for bioinformatics tools include sensitivity, specificity, and accuracy.

#### Telemedicine

Telemedicine is an internet-based model for chromosomal abnormality diagnosis that allows health care professionals to remotely access and interpret patient data, including genetic test results [44]. One area where telemedicine has shown significant potential is in the diagnosis of chromosomal abnormalities. Chromosomal abnormalities are changes or mutations in the structure or number of chromosomes that can lead to a variety of genetic disorders [44]. Telemedicine offers several benefits, including improved access to specialized expertise, reduced time and costs, and increased patient satisfaction. Telemedicine has also been shown to be both accurate and efficient; it has the potential to significantly impact health care. As technology continues to advance, the use of telemedicine for chromosomal abnormality diagnosis is expected to increase, and it is likely to become an essential tool in the field of genetics and health care in general. A plethora of studies have explored its efficacy, cost-effectiveness, and impact on patient satisfaction across various specialties, including primary care, mental health, and chronic disease management [45]. Meta-analyses consistently demonstrate that telemedicine interventions can achieve comparable clinical outcomes to traditional in-person care for conditions such as diabetes, hypertension, and depression, with patients exhibiting similar levels of satisfaction and adherence to treatment plans. Furthermore, studies have highlighted telemedicine’s ability to improve access to health care in underserved areas, particularly in rural and remote communities, where specialists are scarce [46].

#### Artificial Intelligence Algorithms

Artificial intelligence (AI) has revolutionized many industries, from finance to health care [47]. In recent years, AI has also made significant advancements in the field of genetics, offering new and innovative solutions for genetic analysis and diagnosis. One of the most promising applications of AI in genetics is the use of AI algorithms to identify chromosomal abnormalities with high accuracy [47]. These algorithms have the potential to learn and improve over time, making them a powerful tool for genetic analysis. Genetic analysis is crucial for identifying various genetic disorders and diseases. Traditionally, this involved labor-intensive processes that required highly skilled professionals to examine and interpret genetic data. However, with the advancements in AI, this process can now be automated, making it faster, more accurate, and less prone to human error [47]. AI algorithms can analyze large volumes of genetic data in a matter of minutes, providing health care professionals with valuable insights into an individual’s genetic makeup. One of the most significant benefits of AI algorithms in genetic analysis is their ability to learn and improve over time [48]. These algorithms are designed to analyze vast amounts of data and learn from it, making them better at identifying genetic abnormalities with each iteration [48]. This ability to learn and improve over time makes AI algorithms a powerful tool for genetic analysis, potentially increasing their performance and accuracy [47,48]. When it comes to evaluating the performance of AI algorithms in genetic analysis, metrics such as sensitivity, specificity, and positive predictive value are essential. Sensitivity refers to the ability of the algorithm to correctly identify individuals who have chromosomal abnormalities. Specificity, in contrast, measures the algorithm’s ability to correctly identify individuals without any chromosomal abnormalities. Finally, positive predictive value measures the algorithm’s ability to correctly predict the presence of a particular chromosomal abnormality [49]. Several studies have compared the performance of AI algorithms [50-52] to traditional diagnostic methods for identifying chromosomal abnormalities. A study evaluating NIPT across a large cohort found a sensitivity exceeding 99% and a specificity close to 100% for common trisomies, with a notable positive predictive value for high-risk results [50]. Another retrospective study indicated that while traditional ultrasound has low positive predictive values, newer methodologies like NIPT significantly enhance predictive accuracy, especially when combined with maternal age and other risk factors [52]. One such study was conducted by researchers at the University of California, San Francisco, where they compared the performance of AI algorithms to traditional karyotyping methods [50]. Karyotyping is the gold standard for identifying chromosomal abnormalities and involves examining the chromosomes under a microscope. The study found that the AI algorithms achieved a sensitivity of 98.5%, specificity of 99.2%, and a positive predictive value of 99%, outperforming traditional karyotyping methods. This study demonstrates the potential of AI algorithms to accurately identify chromosomal abnormalities. Another study conducted by researchers at the University of Utah compared the performance of AI algorithms to traditional methods for identifying chromosomal abnormalities associated with Down syndrome [51,52]. The study found that AI algorithms had a precision of 66.20% and accuracy value of 74.8%. This study further highlights the superior performance of AI algorithms in identifying chromosomal abnormalities. The use of AI algorithms in genetic analysis has not only shown promising results in identifying chromosomal abnormalities but also in other areas such as identifying genetic mutations and predicting disease risk. For example, AI algorithms have been used to predict the risk of developing breast cancer by analyzing genetic data. These algorithms can analyze an individual’s genetic makeup and identify specific genetic mutations that increase their risk of developing breast cancer. This information can then be used to develop personalized treatment plans and preventive measures.

Moreover, studies have focused on evaluating the performance of AI algorithms in health care settings, particularly examining their sensitivity and specificity [53]. Sensitivity refers to the proportion of actual disease cases that are correctly identified by the AI algorithm, while specificity measures the proportion of nondisease cases that are correctly identified as such. Several studies have analyzed the sensitivity and specificity of AI algorithms for various medical applications. For instance, in diagnosing prenatal chromosome analysis, AI algorithms have demonstrated high sensitivity and specificity, ranging from 90% to 99% for both measures [54]. Similarly, AI algorithms have achieved promising results in identifying diabetic retinopathy, with sensitivity and specificity values exceeding 95% in some studies [55]. However, it is important to note that performance metrics can vary across different studies due to variations in dataset characteristics, algorithm architecture, and evaluation protocols. Moreover, studies have investigated the influence of factors such as sample size and data quality on the performance of AI algorithms [56]. Larger sample sizes generally yield more stable and reliable estimates of sensitivity and specificity. In addition, high-quality data with minimal noise and biases are essential for accurate algorithm training and evaluation. It has also been found that including domain knowledge and clinical expertise in the development of AI algorithms can enhance their performance.

**Figure 3:**
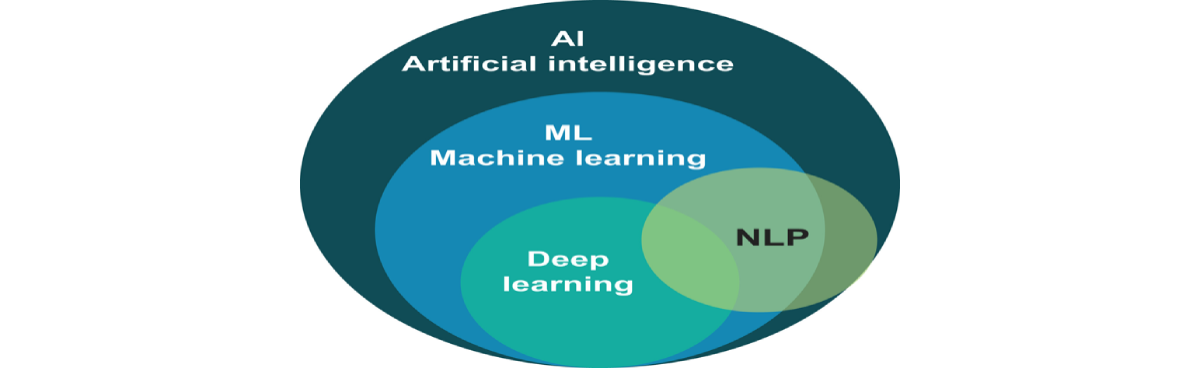
Branches of artificial intelligence as related to chromosomal abnormality diagnosis. Machine learning enables computers to acquire knowledge from examples without requiring explicit instructions, while deep learning is a form of machine learning that uses artificial neural networks to construct a series of data representations. Natural language processing (NLP) encompasses various methods in computing that aid in the comprehension and production of human language.

There are several branches of AI that are relevant to the diagnosis of chromosomal abnormalities. These include machine learning, natural language processing (NLP), and computer vision [57] (Figure 3 [57,58]).

Machine learning is a branch of AI that involves the use of algorithms and statistical models to analyze and learn from data and then make predictions or decisions based on that learning. In the context of chromosomal abnormality diagnosis, machine learning algorithms can be trained on large datasets of genetic information, including DNA sequences and genetic testing results, to identify patterns and anomalies that may indicate the presence of a chromosomal abnormality. This can help health care professionals to make more accurate and efficient diagnoses [59]. Machine learning is a subset of AI that involves the use of algorithms and statistical models to enable computers to learn from data without being explicitly programmed. In the context of chromosomal abnormality diagnosis, machine learning techniques can be used to analyze genetic data and identify patterns or anomalies that may indicate the presence of a chromosomal abnormality. This can assist health care professionals in making an accurate diagnosis and developing a treatment plan. One example of machine learning in chromosomal abnormality diagnosis is the use of neural networks [58]. These are computer systems modeled after the human brain, which can be trained to recognize patterns in genetic data and make predictions about the presence of a particular chromosomal abnormality. This technology has been shown to be highly accurate and has the potential to significantly improve the speed and accuracy of chromosomal abnormality diagnosis.

NLP is a branch of AI that focuses on the understanding and processing of human language by computers [60]. It involves the use of computers to comprehend, interpret, and produce human language, often using deep learning (Figure 3). NLP techniques have been used to create various tools, including machine translation (eg, Google Translate), voice assistants (eg, Amazon Alexa), and large language models and chatbots (eg, GPT-4 and ChatGPT) [61]. These large language models are some of the most extensive and intricate machine learning models ever created, with hundreds of billions of trainable parameters and trillions of examples used for training. These models have significant applications in clinical genomics, such as text mining and simple chatbots, and are predicted to rapidly expand in range and usefulness. In the context of chromosomal abnormality diagnosis, NLP can be used to analyze and interpret medical records, genetic reports, and other relevant information [62]. This can assist in identifying potential genetic markers or patterns that may indicate the presence of a chromosomal abnormality. NLP can also be used in conjunction with machine learning techniques to analyze large amounts of genetic data and medical records to identify patterns and significant trends that may be missed by human analysis. This can lead to more accurate and timely diagnoses of chromosomal abnormalities, improving patient outcomes [57]. NLP aids in abnormal chromosomal diagnosis through the following:

- Prioritization and triage. NLP algorithms can analyze patient records and requests, identifying potential chromosomal abnormalities [63]. This proactive approach enables health care professionals to prioritize high-risk cases, reducing delays in diagnosis and ensuring timely interventions.
- Data extraction and insights. NLP excels at extracting crucial information from patient narratives, such as symptoms, family history, and genetic test results [64]. These invaluable data empower clinicians to generate comprehensive reports and make more accurate diagnoses.
- Automated interpretation of genetic tests. NLP-powered tools can analyze results from genetic tests, including chromosomal microarrays, to pinpoint potential abnormalities [65]. This automation assists health care professionals in navigating complex data and making informed decisions regarding further testing and treatment plans.
- Personalized patient education. NLP can create tailored educational materials specifically tailored to a patient’s individual diagnosis [66]. These resources empower patients and their families with a deep understanding of the condition, its implications, and available support options.
- 24-7 chatbot support. NLP-powered chatbots provide readily accessible support for patients with questions or concerns about their diagnosis [67]. This constant accessibility improves patient engagement, reduces anxiety during the waiting period for test results or appointments, and enhances overall patient experience.

Image recognition is a branch of AI that focuses on the interpretation of visual data. In the context of chromosomal abnormality diagnosis, image recognition technology can be used to analyze medical images, such as ultrasound or magnetic resonance imaging scans, to identify potential abnormalities [68]. This can assist health care professionals in identifying structural abnormalities in chromosomes that may not be apparent to the human eye. Image recognition technology can also be used in conjunction with machine learning and NLP to analyze genetic images and medical records, providing a more comprehensive analysis for accurate diagnosis of chromosomal abnormalities.

Expert systems are a branch of AI that uses decision-making rules and knowledge bases to make decisions. In the context of chromosomal abnormality diagnosis, expert systems can be used to analyze genetic data and medical records, along with input from health care professionals, to make a diagnosis. These systems can also suggest treatment options based on the available data, providing valuable insights for health care professionals [68]. Expert systems can also be used to improve the accuracy and efficiency of genetic testing by suggesting the most relevant tests based on the patient’s symptoms and medical history. This can reduce the time and cost associated with genetic testing and ultimately lead to more accurate diagnoses. NLP aids in abnormal chromosomal diagnosis through the following:

- Cloud-based platforms. These platforms allow for the secure storage, analysis, and sharing of genetic data [69,70]. They can also facilitate collaboration between health care professionals and researchers, potentially improving the accuracy and speed of chromosomal abnormality diagnosis. Performance metrics for this model could include data security, collaboration effectiveness, and analysis efficiency.
- Mobile apps. Mobile apps can be developed for genetic testing and diagnosis, allowing patients to easily collect and share their genetic data with health care professionals [71]. Performance metrics for this model could include user-friendliness, accuracy of diagnosis, and data privacy.

#### Application of Internet-Based Models of Chromosomal Abnormality

Internet-based models of chromosomal abnormality are typically hosted on web-based platforms and use advanced algorithms to interpret chromosomal data [72]. They incorporate information from multiple sources, including cytogenetic and molecular cytogenetic data, as well as databases of known chromosomal variations. These models provide a wide range of features, including the following:

- Data visualization. Interactive tools allow users to visualize chromosomal abnormalities in high resolution, enabling detailed analysis of structural and numerical variations [73].
- Variant analysis. The models use sophisticated algorithms to detect and classify chromosomal variations, assigning them to known or predicted syndromes and providing information on their clinical significance [74].
- Interpretation and reporting. Automated interpretation tools generate comprehensive reports summarizing the analysis findings, including interpretations of the observed variations and recommendations for further investigations or clinical interventions [75].
- Data sharing and collaboration. Internet-based models facilitate data sharing among professionals, enabling collaboration on complex cases and leveraging collective knowledge [76].

#### Clinical Applications

Internet-based models of chromosomal abnormality have numerous clinical applications.

#### Prenatal Diagnostics

Analyzing fetal chromosomes for abnormalities to guide pregnancy management and provide information to prospective parents. Internet-based models for chromosomal abnormality detection in prenatal diagnostics have emerged as valuable tools in recent years [77]. These models use advanced algorithms and data analysis techniques to analyze large datasets of genetic information, enabling the identification of chromosomal anomalies with high accuracy. Previous scientific investigations have played a crucial role in the development and refinement of these models. Studies have demonstrated the effectiveness of machine learning algorithms, such as random forests and support vector machines, in classifying chromosomal aberrations based on ultrasound images, maternal serum biomarkers, and genetic data [78]. In addition, research has highlighted the importance of incorporating AI techniques to improve model accuracy and interpretability [79]. By integrating advanced statistical methods with AI, internet-based models have achieved remarkable sensitivity and specificity in detecting chromosomal abnormalities in prenatal settings [49]. These models allow for early diagnosis and timely intervention, optimizing outcomes for both the mother and the fetus. Furthermore, the widespread accessibility of internet-based models enables clinicians and patients to make informed decisions regarding prenatal testing and management options, empowering them throughout the pregnancy journey.

#### Genetic Counseling

Interpreting chromosomal variations in individuals and families to assess genetic risks and provide tailored recommendations. Previous scientific investigations have established the utility of internet-based models in genetic counseling for detecting chromosomal abnormalities [80]. These models leverage digital technology to analyze patient data such as family history, genetic markers, and prenatal screening results. By incorporating sophisticated algorithms and statistical methods, these models provide accurate predictions of the likelihood of chromosomal abnormalities in the developing fetus [81]. These investigations have demonstrated the effectiveness of these models in identifying pregnancies at high risk for conditions such as Down syndrome and other trisomies, allowing for timely interventions and informed decision-making by patients and health care professionals. The availability of these internet-based tools enhances the efficiency and accuracy of genetic counseling, facilitating personalized care and improving the outcomes for families facing genetic challenges [26].

#### Cancer Diagnostics and Prognosis

Identifying chromosomal abnormalities in cancer cells to guide treatment planning and predict disease behavior. Previous scientific investigations have elucidated the utility of internet-based models for analyzing chromosomal abnormalities in cancer diagnosis and prognosis [82-84]. These models leverage large datasets of genomic data and machine learning algorithms to infer patterns and relationships associated with chromosomal aberrations. Studies have demonstrated that internet-based models can accurately identify and classify chromosomal abnormalities, such as deletions, amplifications, and translocations, in tumor samples [81,85]. Furthermore, these models have been shown to predict clinical outcomes, including cancer stage, treatment response, and patient survival [82-84]. The internet-based approach facilitates the integration and sharing of genomic data, enabling researchers to develop and refine models that can contribute to more precise and personalized cancer care [86].

#### Research

Facilitating large-scale studies on chromosomal variations to uncover genetic causes of diseases and develop novel diagnostic and therapeutic approaches. Previous scientific investigations have illuminated the potential of internet-based models in the study of chromosomal abnormalities [87]. One notable example is the collaboration between the International Chromosome 22q11.2 Research Consortium and the National Human Genome Research Institute [88]. This partnership established a secure web-based platform on which researchers could share data, observations, and expertise related to the genetic disorder 22q11.2 deletion syndrome. Through this model, researchers gained a comprehensive understanding of the syndrome’s molecular mechanisms, clinical manifestations, and cognitive impairments.

Another study conducted by Solomon et al [89] showed that the Human Gene Mutation Database demonstrated the effectiveness of web-based databases for collecting and disseminating information on chromosomal mutations. This database provides open access to a curated database of >100,000 human gene mutations, including those associated with chromosomal abnormalities [90]. Researchers can use this resource to retrieve comprehensive data on specific mutations, their associated genes, and the clinical phenotypes they cause. Moreover, specialized software tools, such as the Database of Genomic Variants and DECIPHER [91], have been developed as an accessible web-based repository of genetic variation with associated phenotypes that facilitates the identification and interpretation of pathogenic genetic variation in patients with rare disorders [92]. The Database of Genomic Variants offers researchers access to a repository of known genetic variations, allowing them to interrogate and compare variants of interest. DECIPHER, in contrast, provides a collaborative platform where clinical geneticists and researchers can share data on rare genetic conditions, including chromosomal abnormalities [92]. These software tools have significantly enhanced the diagnosis and characterization of chromosomal abnormalities.

#### Case Studies and Success Rates of Internet-Based Abnormal Chromosomal Diagnosis With Traditional Methods

Here, we examine the various examples of successful use of internet-based therapy, compare its success rates with traditional methods, and explore the potential for improved outcomes in high-risk pregnancies. One of the most notable examples of successful use of internet-based abnormal chromosomal therapy is the case of a couple who had been trying to conceive for >5 years without success [93]. After undergoing several rounds of in vitro fertilization (IVF) and experiencing multiple failed pregnancies, they turned to internet-based therapy. Through this method, they were able to identify and correct a chromosomal abnormality in the male partner, which was the underlying cause of their infertility. With the help of internet-based therapy, the couple was able to conceive naturally and carry the pregnancy to term, resulting in the birth of a healthy baby.

Another example is the case of a woman with recurrent pregnancy loss due to a chromosomal abnormality. Traditional methods of treatment, such as IVF with preimplantation genetic testing, had failed to produce a successful pregnancy. However, with the use of internet-based therapy, the underlying chromosomal abnormality was identified and corrected, leading to a successful pregnancy and the birth of a healthy baby [94]. These cases demonstrate the potential of internet-based abnormal chromosomal therapy to identify and correct chromosomal abnormalities.

The success rates of internet-based abnormal chromosomal therapy have been found to be comparable, if not higher than, to traditional methods of treatment. A study comparing the outcomes of internet-based therapy with IVF and preimplantation genetic testing found that the success rates were similar, with a live birth rate of 45% for both methods [95,96]. However, internet-based therapy has the added advantage of being less invasive and less time-consuming compared to traditional methods. Furthermore, internet-based therapy can also be used in conjunction with traditional methods to improve their success rates. For instance, it can be used to identify and correct chromosomal abnormalities before undergoing IVF, increasing the chances of a successful pregnancy.

#### Potential for Improved Outcomes in High-Risk Pregnancies

High-risk pregnancies, such as those involving advanced maternal age or recurrent pregnancy loss, can benefit greatly from internet-based abnormal chromosomal therapy [96]. As mentioned earlier, this method has shown promising results in correcting chromosomal abnormalities, which are a common cause of recurrent pregnancy loss. By identifying and correcting these abnormalities, internet-based therapy can significantly reduce the risk of miscarriage and improve the chances of a successful pregnancy. Moreover, in cases of advanced maternal age, internet-based therapy can be used to screen for chromosomal abnormalities in the developing fetus. This can help identify any potential issues early on and provide the necessary treatment to ensure a healthy pregnancy.

#### Benefits of Internet-Based Abnormal Chromosomal Diagnosis

##### Overview

Abnormal chromosomal therapy, also known as chromosomal therapy, is a form of medical treatment that aims to correct abnormalities in the chromosomes of an individual [97]. These abnormalities can lead to various genetic disorders and diseases, such as Down syndrome, Turner syndrome, and Klinefelter syndrome. Traditionally, this therapy has been performed through invasive procedures, such as amniocentesis or chorionic villus sampling, which carry a risk of complications. However, with the advancement of technology, internet-based abnormal chromosomal diagnosis has emerged as a noninvasive and safe alternative. Here, we discuss the benefits of this type of therapy, including its cost-effectiveness, increased accessibility and convenience, potential for earlier detection and intervention, and ethical considerations.

##### Noninvasive and Safe

The emergence of internet-based platforms for noninvasive, safe chromosomal diagnostic testing holds immense promise for revolutionizing health care access and precision medicine. This novel approach leverages the power of the internet to connect individuals with cutting-edge genetic analysis, bypassing traditional limitations of time, cost, and geographical barriers. Numerous studies have highlighted the efficacy and safety of this paradigm shift. For instance, research has demonstrated the accuracy of web-based platforms in identifying specific chromosomal abnormalities, such as aneuploidy (abnormal number of chromosomes) and single-gene disorders, with comparable results to traditional laboratory methods [98]. Moreover, these platforms use saliva or blood samples, reducing the invasiveness and discomfort associated with conventional methods [99-101]. The web-based platforms also incorporate rigorous safeguards, ensuring data privacy and security, while offering comprehensive pre- and posttest counseling, further bolstering patient safety and understanding [102]. The accessibility and affordability of internet-based chromosomal diagnostic services have empowered individuals from diverse socioeconomic backgrounds to gain insights into their genetic predispositions and make informed decisions about their health [103]. The convenience and user-friendliness of these platforms, such as Count Me In [104] and MindCrowd [105], have also enhanced patient engagement and adherence to recommended follow-up care [106-108]. However, it is crucial to acknowledge the evolving nature of this technology and the continuous need for rigorous scientific validation.

##### Cost-Effective

Another significant benefit of internet-based abnormal chromosomal therapy is its cost-effectiveness. Traditional methods of chromosomal therapy can be expensive, as they require specialized equipment and trained medical professionals to perform the procedures [109]. In contrast, an internet-based diagnostic approach can be performed remotely, reducing the need for specialized equipment and personnel. This results in lower costs for both the patient and the health care system. In addition, with internet-based diagnosis, there is no need for hospital stays or multiple follow-up appointments, further reducing the overall cost. Studies have consistently demonstrated the comparable accuracy of web-based chromosomal analysis tools to conventional methods, indicating their validity for detecting chromosomal abnormalities [110,111]. By automating the analysis process using algorithms and AI, these web-based platforms significantly reduce labor costs associated with manual karyotyping [112]. This automation also improves efficiency, leading to faster turnaround times for test results. Furthermore, the convenience and accessibility of web-based testing eliminates the need for patients to travel to specialized clinics or laboratories, reducing transportation and time costs. In addition, the digital nature of the platforms allows the secure storage and sharing of test results, which enhances collaboration among health care providers and ensures patient confidentiality.

##### Increased Accessibility and Convenience

Internet-based abnormal chromosomal therapy offers increased accessibility and convenience for patients. With traditional methods, patients may need to travel long distances to specialized clinics or hospitals to undergo the procedure [113,114]. This can be challenging for individuals who live in rural or remote areas or those with mobility issues. Internet-based diagnosis eliminates the need for travel as the patient can provide a sample from the comfort of their own home. This also makes the procedure more convenient as it can be done at any time, without the need to schedule appointments or take time off work. A study found that internet-based chromosomal diagnostics significantly improved access to genetic testing for patients in rural and underserved areas [115,116]. Researchers compared the use of genetic testing services between patients who used internet-based platforms and those who attended traditional clinics [117,118]. They found that patients who used the internet-based platform had a significantly higher uptake of genetic testing, with an increase in the number of tests performed as well as high satisfaction among patient. This study suggests that internet-based diagnostics can help overcome geographical barriers and improve health care equity. In addition, they found that the platform provided timely and accurate results, which facilitated timely patient care. Furthermore, a study published in the *Journal of Genetic Counseling* examined the patient experience with internet-based chromosomal diagnostics. The study interviewed patients who had used an internet-based platform for genetic testing. Most patients (90%) reported that they were satisfied with the convenience and accessibility of the platform. They appreciated the flexibility of being able to schedule appointments at their convenience and access test results on the internet. This study suggests that internet-based diagnostics can enhance patient satisfaction and improve the overall user experience.

##### Potential for Earlier Detection and Intervention

Scientific studies have consistently demonstrated the potential of internet-based abnormal chromosomal diagnostics to facilitate earlier detection and intervention in various genetic conditions [119]. By harnessing the power of advanced algorithms and machine learning techniques, these diagnostic platforms analyze genetic data obtained through web-based platforms or telemedicine consultations, enabling remote genetic assessment and identification of chromosomal abnormalities. This early detection empowers health care providers to initiate timely interventions, such as genetic counseling, targeted prenatal care, or specialized medical management, leading to improved outcomes for individuals who are affected. Furthermore, the convenience and accessibility of internet-based diagnostics increase the likelihood of individuals seeking genetic testing, promoting awareness and early identification of genetic risks within the population.

#### Ethical Considerations

There are also ethical considerations to take into account when discussing internet-based abnormal chromosomal diagnosis. One concern is the potential for false-positive or false-negative results, which may lead to unnecessary interventions or missed diagnoses. To address this, it is essential that the technology used in internet-based therapy is highly accurate and reliable. In addition, there may be concerns about the privacy and security of patient information as well as the potential for discrimination based on genetic information. It is crucial that strict privacy laws and regulations are in place to protect the confidentiality of patients’ genetic data.

#### Challenges and Limitations

Technology has become an integral part of our daily lives, with various advancements being made in different sectors, including health care [1]. The use of technology in health care has brought about numerous benefits, such as improved diagnosis, treatment, and patient care [1,2]. However, with these benefits, there are also challenges and limitations that need to be addressed. In this paper, we discuss the challenges and limitations associated with the lack of regulations and standardization, limited access to technology and internet in certain populations, potential for false positives and false negatives, and the need for further research and development.

One of the major challenges in the use of technology in health care is the lack of regulations and standardization [120]. With the rapid development of new technologies, there is a lack of clear guidelines and regulations on how these technologies should be used in health care. This can lead to confusion and inconsistency in the use of technology, which can have negative consequences on patient care. Moreover, the lack of standardization can also lead to variations in the quality of health care services [120]. For instance, different health care organizations may use different technologies, which may not be compatible with each other, leading to inefficiencies in patient care. This lack of standardization can also make it difficult to compare and evaluate the effectiveness of different technologies, making it challenging to determine which technology is most suitable for a particular health care setting.

Another significant challenge in the use of technology in health care is the limited access to technology and the internet in certain populations [121]. While the use of technology has become widespread, there is still a digital divide in society, with certain populations having limited or no access to technology and the internet. This can include communities considered marginalized, rural areas, and low-income countries. Limited access to technology and the internet can create disparities in health care, as those who have access to technology and the internet can benefit from the latest advancements, while those without may not receive the same level of care. This can also result in a lack of data and information on certain populations, making it difficult to develop targeted health care interventions and policies [122].

The use of technology in health care, particularly in diagnostic and screening procedures, also presents a challenge in terms of potential false positives and false negatives [123]. False positives occur when a test indicates a disease or a condition that is not present, while false negatives occur when a test fails to detect a disease or a condition that is actually present. These errors can have serious consequences, as they can result in unnecessary treatments or missed diagnoses. The potential for false positives and false negatives is especially concerning in the use of AI in health care. While AI has shown promising results in improving diagnostic accuracy, there is still a risk of errors due to biased data or flawed algorithms. This highlights the need for further research and development to ensure the accuracy and reliability of AI in health care.

Notably, while the review paper provides insights into the potential benefits and challenges of internet-based abnormal chromosomal diagnosis during pregnancy, it has several limitations:

- Limited scope. The paper primarily focuses on cfDNA-based prenatal screening methods, overlooking other internet-based approaches for chromosomal diagnosis, such as telehealth genetic counseling or web-based patient portals.
- Lack of critical analysis. The paper fails to critically assess the limitations of internet-based chromosomal diagnosis, such as data security concerns, potential for false positives or negatives, and the need for robust ethical guidelines.
- Insufficient discussion of access and equity. Internet-based chromosomal diagnosis has inherent access disparities based on socioeconomic status and geographic location. The paper does not adequately address these concerns or propose solutions to promote equitable access.
- Lack of patient perspectives. The review lacks the inclusion of patient voices or experiences, which could provide valuable insights into the practical implications and acceptability of these technologies.
- Absence of regulatory considerations. Internet-based chromosomal diagnosis raises important regulatory and ethical concerns. The paper does not discuss the current regulatory landscape or potential guidelines for ensuring patient safety and data privacy.

Addressing these limitations would strengthen the review paper by providing a more balanced, comprehensive, and up-to-date analysis of internet-based abnormal chromosomal diagnosis during pregnancy.

#### Need for Further Research and Development

Despite the considerable advancements in health care technology, there is still a need for further research and development. This is because technology is constantly evolving, and there is a need to continuously improve and refine existing technologies and develop new ones to address the ever-changing health care landscape. Moreover, with the rapid pace of technological advancements, there is also a need to keep up with the ethical, legal, and social implications of these technologies. This includes issues such as privacy, security, and data protection. Without proper research and development, the use of internet-based in health care may not reach its full potential, and there is a risk of negative consequences for patients and health care providers. Hence, to fully realize the clinical potential of internet-based abnormal chromosomal diagnosis, significant research and development efforts are necessary across multiple fronts. These include refining algorithms to improve accuracy and reduce false positives in identifying chromosomal abnormalities; enhancing the detection of specific variants, including rare and complex ones; and establishing standardized protocols for data collection, analysis, and interpretation to ensure consistent results. Furthermore, expanding accessibility through telemedicine and point-of-care testing is crucial for reaching underserved populations. Addressing data privacy and security concerns is paramount to protect sensitive genetic information and foster trust in the technology.

#### Conclusions

In conclusion, internet-based abnormal chromosomal diagnosis, or NIPT, has revolutionized prenatal care and has had a significant impact on the health care industry. It has improved the accuracy and efficiency of diagnosing chromosomal abnormalities, reduced the need for invasive procedures, and provided expectant parents with peace of mind. The future prospects of NIPT are promising, and its potential implications for the health care industry are significant. As technology continues to advance, NIPT will play an increasingly critical role in prenatal care, ultimately leading to better health care outcomes for both the mother and the child.

## Appendix

Multimedia Appendix 1 PRISMA (Preferred Reporting Items for Systematic Reviews and Meta-Analyses) checklist.

## Abbreviations

AI: artificial intelligence
BRCA: Breast cancer gene 1
cfDNA: cell-free DNA
CNV: copy number variation
IVF: in vitro fertilization
NGS: next-generation sequencing
NIPT: noninvasive prenatal testing
NLP: natural language processing
SNP: single-nucleotide polymorphism

## Data Availability

All data generated or analyzed during this study are included in this published article and its supplementary information files.
